# Body-weight supported treadmill or total body recumbent stepper for mobility-adapted cardiopulmonary exercise testing in multiple sclerosis patients with varying disability

**DOI:** 10.3389/fresc.2026.1731215

**Published:** 2026-03-05

**Authors:** Saman Hadjizadeh Anvar, Liam P. Kelly, Caitlin Newell, Lynsey Alcock, Hamidreza Barzegarpoor, Michelle Ploughman

**Affiliations:** Faculty of Medicine, Memorial University of Newfoundland, St. John’s, NL, Canada

**Keywords:** aerobic capacity, cardiopulmonary exercise test, cardiorespiratory, cardiovascular, disability, fitness, multiple sclerosis, muscle fatigue

## Abstract

**Background:**

Accurate exercise prescription in persons with multiple sclerosis (MS) depends on cardiopulmonary exercise testing (CPET). Obtaining an accurate VO_2max_ can be challenging when patients experience lower limb impairment and fatigue that can change over time.

**Objective:**

We sought the optimal adapted device to achieve a maximal CPET among persons with MS.

**Methods:**

In a randomized crossover trial with within-subject, repeated measures design, clinic-referred persons with MS (*n* = 10) with three-month stability, no exercise obstruction, ability to walk with or without assistance, and sex- and age-matched (±3 years) Controls (*n* = 7) were recruited by convenience sampling. Participants performed CPET on body weight-supported treadmill (BWST) and total body recumbent stepper (TBRS). We collected standard aerobic metrics, including $\dot{V}O_{2\max}$, % normative values for $\dot{V}O_{2\max}$ (%$\dot{V}O_{2\max}$), heart rate maximum (HR_max_), age-predicted HR_max_, and Respiratory Exchange Ratio.

**Results:**

MS patients, regardless of disability severity, achieved similar $\dot{V}O_{2\max}$ values (mL·min^−1^·kg^−1^) on the TBRS and BWST. Control participants obtained higher values on BWST (40.27 ± 7.6 vs. 34.32 ± 7.1, *p* < 0.001). MS patients more consistently met the criteria for maximal CPET using the TBRS (*n* = 9/10 vs. 6/10 on BWST).

**Conclusions:**

$\dot{V}O_{2\max}$ was similar between devices among MS patients. On BWST, they achieved lower $\dot{V}O_{2\max}$ values compared to Controls. MS patients more successfully achieved the primary criterion, VO_2_ plateau (≤150 ml/min^−1^), using TBRS. Additionally, TBRS permitted persons with mobility disability to achieve more criteria for a maximum CPET. Our results suggest that CPET using BWST, being reliant on the lower body, likely disadvantages MS patients, especially those with mobility disability.

## Introduction

Multiple sclerosis (MS) is among the most prevalent neurological disorders among young and middle-aged people (1). Adopting an inactive lifestyle (2) results in inactivity-related health problems among persons with MS (PwMS), such as osteoporosis, cardiovascular disease, fatigue, and depression (3). Aerobic capacity is a crucial health (4) and performance marker (5) (6),, and PwMS exhibit lower values of maximal aerobic power when compared to age and sex-matched healthy adults (7). Importantly, lower risk of cardiovascular disease (6), better walking performance (8), faster cognitive processing speed (9), and enhanced neuroprotection and brain health (10), have all been associated with greater aerobic capacity in MS (6). Testing aerobic fitness accurately, as a metric to calculate personalized exercise intensity and as a study outcome measure, is recommended (6).

The gold standard assessment of aerobic fitness measures the maximum rate of oxygen consumption ($\dot{V}O_{2\max}$), in relation to the rate of carbon dioxide ($\dot{V}CO_{2}$) produced during a progressive maximal cardiopulmonary exercise test (CPET). CPET is feasible and safe among persons with MS (PwMS) (11–13). While several different exercise testing modalities (e.g., treadmill, bicycle ergometer, recumbent stepper) have been used to assess aerobic capacity, there is no evidence-based consensus regarding the best instrument that should preferentially be used in people having mobility disability (13).

Weakness and fatigue of the limbs can limit the ability to reach maximum aerobic capacity during a CPET (14, 15). Additionally, leg weakness and fear of falling could impede maximal effort, even when using treadmills with overhead harness support (16). Although yet to be determined, modalities that permit the user to exercise in a seated position, such as total body recumbent steppers (TBRS), may permit CPET metrics that represent true cardiopulmonary capacity, less influenced by leg impairments (17). Obtaining stable and representative CPET values would be especially useful in longitudinal studies, as there may be an emergence of MS-related relapses or accumulation of disability within an individual.

Investigators have tested several adapted and non-adapted methods for CPET among PwMS, including bicycle ergometers, arm ergometers, and TBRS (11, 18). For instance, Pilutti and colleagues compared arm ergometers to TBRS, and reported higher peak aerobic capacity achieved on the TBRS, especially among people with low levels of disability and higher fitness levels (19). Although useful in determining optimal CPET methodology, the authors did not provide comprehensive reporting of criteria necessary for $\dot{V}O_{2\max}$ achievement (19, 20), so whether maximum CPET was achieved cannot be assured. The researchers recommended the use of a TBRS over the arm ergometer for CPET in PwMS. Similarly, evidence from our laboratory, testing two common adapted CPET methods (TBRS and Body Weight Supported Treadmill-BWST) suggested less fatigue of the soleus muscle when the workload was distributed between four limbs (using a TBRS) rather than two (using a BWST) (21).

We aimed to compare CPET metrics using two adapted modalities (BWST and TBRS) among PwMS with varying mobility disability and matched controls. Metabolic parameters ($\dot{V}O_{2\max}$, % normative values for $\dot{V}O_{2\max}$-% $\dot{V}O_{2\max}$, heart rate maximum-HR_max_, and age-predicted HR_max_) were obtained along with the ability of participants to meet criteria for achieving a maximum CPET (22). We also aimed to preliminarily investigate whether the level of disability influenced the ability to achieve $\dot{V}O_{2\max}$ indicators. We hypothesized that: a) Controls would achieve similar $\dot{V}O_{2\max}$ on the BWST and TBRS; b) PwMS would achieve higher values and be more likely to meet $\dot{V}O_{2\max}$ criteria on TBRS, especially for those having greater mobility disability.

## Methods

### Participants

We recruited a convenience sample of persons who attended an MS clinic and had participated in previous studies (23, 24). Ten PwMS agreed to participate in this sub-study. To be included, we confirmed: a) MS diagnosis using McDonald criteria (25), b) no relapses/stable during the previous three months, c) a negative Physical Activity Readiness Questionnaire (PAR-Q) screening (26), d) no musculoskeletal obstruction to exercise, and e) scoring greater than 24 on Montreal Cognitive Assessment (MoCA) (27), f) ability to walk at least 10 m with or without assistance. We included a broad EDSS range to reflect the clinical population for whom adapted CPET is used and to allow examination of how disability levels interact with modality performance through planned subgroup analyses. Sex and age-matched Controls (±3 years) were recruited by convenience sampling from an existing cohort participating in a separate study conducted in our laboratory. Competitive athletes were excluded (28). After obtaining written informed consent, sex, age, height, weight, Expanded Disability Status Scale (EDSS), type of MS (relapsing-remitting-RRMS, secondary progressive-SPMS, or primary progressive-PPMS), medications, and co-morbid conditions were recorded. This study was approved by the Health Research Ethics Board (HREB) of [the name of the HREB University was omitted to maintain anonymity during peer review] (Approval Ref. #: 14.102).

### Experimental design

In a randomized crossover trial with within-subject repeated measures design, participants performed the CPET on the BWST and TBRS, 7–10 days apart, at the same time of day, with five minutes each of warm-up and cool-down. The order of testing (BWST vs. TBRS) was randomized using a computer-generated sequence before assessment. Participants were required to avoid food, caffeine, and intense exercise for at least two, six, and 12 h, respectively, before the tests (20).

### Interventions

#### Total body recumbent stepper (TBRS)

Using NuSTEP T4r Recumbent Stepper (NuStep Inc, Ann Arbor, MI) in a seated position (12, 29), participants were required to maintain a speed of 80 strides per minute. The resistance [1–10, beginning at level 3 = 25 watts (30)] was increased by one unit (=15 watts) every two minutes (30). If the participant did not reach exhaustion at the highest resistance level (Level 10), the speed (strides per minute) was increased by ten every two minutes until volitional exhaustion terminated the test.

#### Body weight support treadmill (BWST)

A rehabilitation treadmill (Sport Art T625M/T52 MD-Rehabilitation Commercial Treadmill, USA) was used with an overhead support harness at 10% of body weight. CPET started at a self-selected speed for two minutes with a %0 treadmill grade. While keeping a constant speed, the grade was increased by 2.5% every two minutes until the grade reached 10%. Reaching grade 10, the speed increased by 0.05 m/s every two minutes until volitional exhaustion.

After calibration, indirect calorimetry (Moxus Metabolic Systems, AEI Technologies, Inc., Pittsburgh, Pennsylvania, USA) was used to measure the rate of oxygen consumption ($\dot{V}O_{2}$), carbon dioxide production ($\dot{V}{\mathrm{CO}}_{2}$), and HR (HR: Polar V800, Polar Electro Oy, Professorintie 5, FI−90,440, Kempele, Finland).

All data were obtained through direct cardiopulmonary exercise testing (CPET) performed by trained staff using standardized protocols. We recorded criteria for the termination of the CPET: (i) RPE >7/10; (ii) no HR or $\dot{V}$ O_2_ increase despite increases in workload; (iii) inability to maintain the required workload or speed. Achievement of maximal oxygen consumption was assessed based on the attainment of 2 or more of the following criteria: (a) plateau in $\dot{V}O_{2}$ (≤150 ml/min^−1^), (b) RER ≥1.1; and/or (c) HR_max_ ± 10 beats per minute (bpm) of age-predicted HR_max_ based on the following equation: 206.9—(0.67 * age) or 164—(0.7 * age) if β-blockers prescribed (29, 31), (d) volitional exhaustion. Relative $\dot{V}O_{2\max}$ was calculated with the highest absolute $\dot{V}O_{2}$ divided by the body weight and reported as ml.min^−1^.kg^−1^. $\dot{V}O_{2\max}$ was also converted to normative values (%$\dot{V}O_{2\max}$) based on the American College of Sports Medicine (ACSM) Guidelines for Exercise Testing and Prescription (22). The time to test completion was also recorded in minutes.

We used the STROBE reporting guideline (32) to draft this manuscript, and the STROBE reporting checklist when editing, included in Supplementary Material S1.

### Statistical analyses

Data were analyzed using SPSS version 27 software. Normality (Shapiro–Wilk test), homogeneity of variance (Levene's test), and sphericity (Mauchly test) assumptions were assessed and confirmed. Greenhouse-Geisser epsilon was reported in the case of sphericity violation. Repeated measures ANOVA tests were used to detect any potential differences between MS and Controls in any of the dependent variables ($\dot{V}O_{2\max}$, % $\dot{V}O_{2\max}$, HR_max_, age-predicted HR_max_) on both modalities (TBRS, BWST), and the interaction effect- modality (TBRS vs. BWST) * Group (MS vs. Control). To examine the influence of disability severity on CPET outcomes, PwMS were stratified for subgroup analyses based on EDSS scores (EDSS ≤2 vs. EDSS >2). This cut-off was selected to reflect the emergence of mobility-related functional impairment reported in prior literature (33). One-way ANOVA tests were also used to compare disability level groups (EDSS >2 and ≤2) with Control on BWST and TBRS. Chi-square test was used to compare the number of exercise criteria achievement on TBRS and BWST- MS (EDSS >2& ≤2 vs. Control). Each participant's achievement of fitness criteria on each modality was recorded as “Yes” or “No.” A significance level of *α* = 0.05 was chosen to assess the statistical significance of all testing variables. Effect sizes were reported as partial eta-squared (*µ*^2^), with 0.02, 0.13, and 0.26 considered small, medium, and large effects, respectively (34).

## Results

### Participants

The median (Q1-Q3) EDSS score for PwMS was 2.25 (0.75–3.1), with a range of 0–6.0, with half scoring 2.5 (minimal disability) or greater. On average, the PwMS ranged in age from 27 to 63 years. Groups did not differ in age (*p* = 0.9), or BMI (*p* = 0.1). Individual-level and summarized demographic and metabolic data are provided in Tables 1, 2, respectively. Controls did not differ in time to test completion between BWST and TBRS (*p* = 0.5). However, PwMS exercised about 9 min longer on BWST compared to TBRS (*p* < 0.001).

**Table 1:** Participant demographics.

Group	ID	Sex	Age range	BMI (kg/m^2^)	EDSS	MS Type	MS medication
MS Patients	1	Female	51–55	35.4	6.0	PPMS	MFS^b^
	2	Male	51–55	28.6	3.5	RRMS	DMD^a^
	3	Female	56–60	21.9	3.0	RRMS	DMD^a^
	4	Female	56–60	27.3	3.0	RRMS	DMD^a^
	5	Female	46–50	31.7	2.5	RRMS	DMD^a^
	6	Female	36–40	20.5	2.0	RRMS	DMD^a^
	7	Female	26–30	19.2	1.0	RRMS	DMD^a^
	8	Female	61–65	23.5	1.0	RRMS	DMD^a^
	9	Female	46–50	24.5	0	RRMS	None
	10	Female	41–45	34.7	0	RRMS	None
		Mean ± SD:	49.2 ± 10.8	26.7 ± 5.7	2.2 ± 1.8	N/A	N/A
Healthy Controls	1	Female	51–55	19.6	None	None	None
	2	Female	56–60	21.9	None	None	None
	3	Female	41–45	22.7	None	None	None
	4	Female	46–50	24.4	None	None	None
	5	Male	51–55	25.2	None	None	None
	6	Female	26–30	24.6	None	None	None
	7	Female	61–65	24.8	None	None	None
		Mean ± SD:	50.3 ± 11.3	23.3 ± 2.03	N/A	N/A	N/A

EDSS, expanded disability status scale; RRMS, relapsing remitting MS; PPMS, primary progressive MS.

^a^ Disease-modifying drug.

^b^ Medication for spasticity.

**Table 2:** Individual metabolic parameters achieved.

Group	ID	V̇O_2max_ (mL*min^−̇^kg^−1^)		RER (VCO_2_/VO_2_)		HRmax (bpm)		%$\dot{V}O_{2\max}$		%HRmax		Rate of perceived exertion (RPE)		Time to test completion	
		TBRS	BWST	TBRS	BWST	TBRS	BWST	TBRS	BWST	TBRS	BWST	TBRS	BWST	TBRS	BWST
MS Patients (EDSS >2)	1	12.26	10.81	0.89	0.99	104	104	<1	<1	61	61	7	6	13:16	14:17
	2	36.50	24.97	1.13	0.99	187	147	50	5	109	86	10	9	14:33	21:58
	3	28.97	19.89	1.06	0.99	144	120	40	1	87	72	8	8	11:57	24:01
	4^a^	20.58	21.63	1.20	1.06	143	140	10	10	117	115	9	10	12:39	29:51
	5	14.48	16.72	1.03	1.04	123	149	<1	<1	71	86	10	9	13:25	17:38
Mean ± SD:		22.5 ± 10.1	**18.8 ± 5.4** ^c^ ^,^ ^d^	1.06 ± 0.1	1.01 ± 0.0	140 ± 31	**132 ± 19** ^d^	**20 ± 24** ^c^	**3 ± 4** ^c^ ^,^ ^d^	89 ± 24	84 ± 20	8.8	8.4	**12:9 ± 0.4** ^b^	**21:3 ± 2.6** ^b^
MS Patients (EDSS ≤2)	6	35.55	37.41	1.21	1.08	159	170	65	70	89	95	9	10	16:28	20:58
	7	35.69	34.16	1.17	1.15	193	197	40	35	103	105	10	10	14:18	30:47
	8	25.76	25.81	1.07	1.11	129	129	35	35	79	79	9	10	13:16	18:54
	9	30.78	26.47	1.21	1.03	183	186	35	15	105	107	10	10	15:07	34:05
	10	24.70	24.51	1.13	0.97	172	176	5	5	97	99	10	9	19:07	17:43
Mean ± SD:		30.5 ± 5.2	**29.6 ± 5.7** ^c^ ^,^ ^d^	1.16 ± 0.1	1.07 ± 0.1	167 ± 25	172 ± 26	**36 ± 21** ^c^	**32 ± 25** ^c^ ^,^ ^d^	94 ± 11	97 ± 11	9.6	9.8	**15.5 ± 1.01** ^b^	**24:2 ± 3.3** ^b^
Healthy controls	1	36.26	42.98	0.98	0.96	176	187	85	99	104	111	7	9	14:34	13:37
	2	33.61	40.58	1.06	1.03	172	180	85	99	104	109	10	10	17:00	17:16
	3	40.95	47.93	1.15	0.97	176	172	85	95	99	97	10	10	16:23	13:09
	4	25.63	33.63	1.13	1.13	153	168	20	70	89	97	10	9	16:30	17:09
	5	45.42	51.09	1.24	0.99	170	176	85	95	99	103	10	9	22:23	36:36
	6	29.21	34.41	1.23	1.14	170	184	15	45	92	99	7	10	16:49	14:55
	7	29.14	31.30	1.06	1.12	171	173	60	75	104	106	7	7	16:42	20:38
Mean ± SD:		**34.3 ± 7.1** ^b^	**40.2 ± 7.6** **^b^** ^,c^	1.12 ± 0.1	1.05 ± 0.1	170 ± 7.8	**177 ± 6.8** ^d^	**62 ± 32** **^b^** ^,c^	**83 ± 20** ^b^ ^,^ ^c^ ^,^ ^d^	99 ± 6.3	103 ± 5.5	8.7	9.1	17 ± 0.9	18.8 ± 3.0

MS, multiple sclerosis; EDSS, expanded disability status scale; RER, respiratory exchange ratio; CO2, volume of dioxide oxygen; O2, volume of oxygen; HRmax, maximum heart rate (beats per minute (bpm); % O2max, % normative values for O2max; %HRmax, % age-predicted heart rate maximum.

Bold values indicate statistically significant differences.

^a^ Participant on beta-blocker medication.

^b^ Significant differences between TBRS and BWST (*p* < 0.05).

^c^ Significant differences between CONTROL and MS (*p* < 0.05).

^d^ Significant differences between the MS severity (EDSS <2, EDSS >2) and CON.

### Metabolic parameters

#### V̇O_2max_

**Figure 1:**
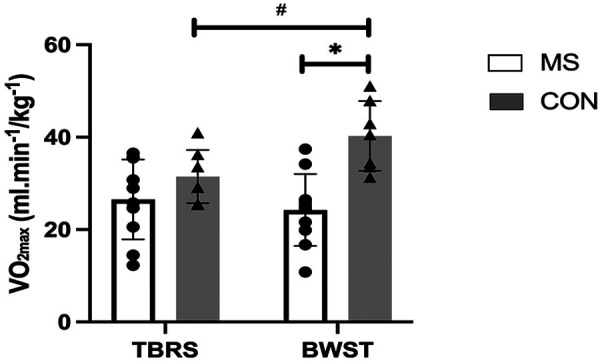
Comparison of cardiorespiratory fitness ($\dot{V}O_{2\max}$) between people with multiple sclerosis (MS) and healthy controls when both groups performed CPET on body weight supported treadmill (BWST) and total-body recumbent stepper (TBRS). Data are presented as mean ± SD. Each symbol represents one participant (MS: *n* = 10; CON: *n* = 7). The Control group achieved a significantly higher $\dot{V}O_{2\max}$ on the BWST (#; *p* < 0.001); Multiple Sclerosis participants had a significantly lower $\dot{V}O_{2\max}$ than the Control on the BWST (*; *p* < 0.001), but not on the TBRS. Statistical Analyses were performed using two-way ANOVA with factors Group (MS vs. CON) and Test (TBRS vs. BWST).

**Figure 2:**
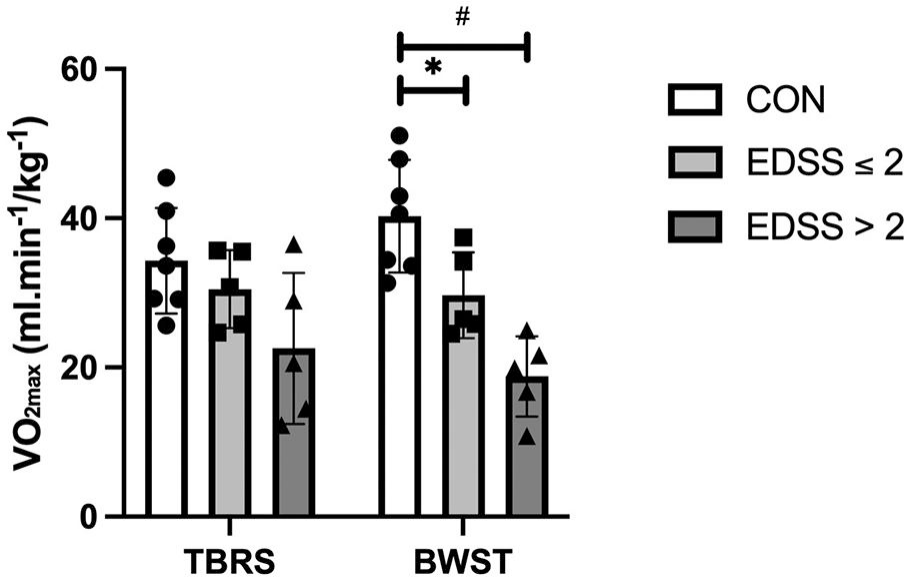
Comparison of cardiorespiratory fitness ($\dot{V}O_{2\max}$) between people with multiple sclerosis (MS) having different MS-severities (EDSS ≤2, EDSS >2) and healthy controls when performed CPET on body weight supported treadmill (BWST) and total-body recumbent stepper (TBRS). Data are presented as mean ± SD. Each symbol represents one participant (EDSS >2: *n* = 5; EDSS ≤2: *n* = 5; CON: *n* = 7). Participants with higher Multiple Sclerosis severity (EDSS >2) had significantly lower values than the healthy controls on BWST (#, *p* < 0.001, 95% C.I. 11.1, 31.7). People with lower severity of MS (EDSS ≤2) had also lower values than the healthy controls on BWST (*, *p* = 0.04, 95% C.I. 0.27, 20.9). Statistical analyses were performed using two-way ANOVA, followed by Bonferroni *post-hoc* comparisons.

**Figure 3:**
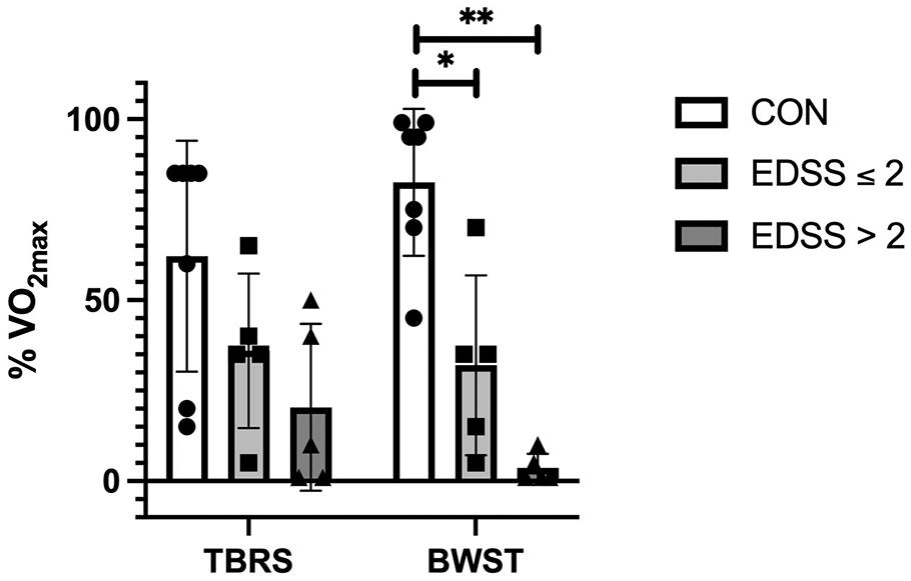
Comparison of % $\dot{V}O_{2\max}$ (% normative) values between people with multiple sclerosis (MS) and healthy controls when both groups performed CPET on body weight supported treadmill (BWST) and total-body recumbent stepper (TBRS). Data are presented as mean ± SD. Each symbol represents one participant (MS: *n* = 10; CON: *n* = 7). Healthy controls had significantly higher values on BWST, compared to TBRS (#, *p* = 0.002). People with MS had lower values than the controls on both BWST (**, *p* < 0.001) and TBRS (*, *p* = 0.02) [F _(1, 15)_ = 13.9, *p* = 0.002, *µ*^2^ = 0.48]. Statistical Analyses were performed using two-way ANOVA with factors Group (MS vs. CON) and Test (TBRS vs. BWST).

**Figure 4:**
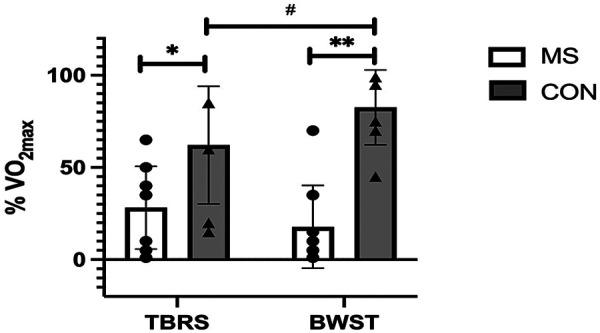
Comparison of % $\dot{V}O_{2\max}$ (% normative) values between people with multiple sclerosis having different MS-severities (EDSS ≤2; EDSS >2) and healthy controls when all groups performed CPET on body weight supported treadmill (BWST) and total-body recumbent stepper (TBRS). Data are presented as mean ± SD. Each symbol represents one participant (EDSS >2: *n* = 5; EDSS ≤2: *n* = 5; CON: *n* = 7). The Multiple Sclerosis participants with higher Multiple Sclerosis severity (EDSS >2) had significantly lower values than the controls on BWST (**, *p* < 0.001, 95% C.I. 49.2, 109.5). People with lower Multiple Sclerosis severity (EDSS ≤2) had significantly lower values than the controls on BWST (*, *p* = 0.001, 95% C.I. 20.4, 80.7). Statistical analyses were performed using two-way ANOVA, followed by Bonferroni *post-hoc* comparisons.

As expected, the Control Group's $\dot{V}O_{2\max}$ was significantly higher than PwMS overall, as a main effect was observed for the group (MS vs. Control) with a large effect size [F (_1, 15_) = 10.0, *p* = 0.006, *µ*^2^ = 0.4; Table 3; Figure 1]. Although PwMS achieved similar $\dot{V}O_{2\max}$ values on both the BWST and TBRS, the Control group achieved higher $\dot{V}O_{2\max}$ on the BWST compared to the TBRS [Interaction effect, F _(1,15)_ = 19.3, *p* < 0.001, *µ*^2^ = 0.5] (Figure 1). PwMS had lower $\dot{V}O_{2\max}$ than Controls on the BWST (24.2 ± 7.8 vs. 40.2 ± 7.6 mL·min^−1^·kg^−1^) but not on the TBRS (26.5 ± 8.7 vs. 34.3 ± 7.1 mL·min^−1^·kg^−1^). A preliminary analysis suggested differences between the Controls and MS disability subgroups were more apparent when using BWST, likely because the Controls achieved higher $\dot{V}O_{2\max}$ on BWST. (Table 3; Figure 2). After $\dot{V}O_{2\max}$ values were transformed into % normative values for age and sex (Table 3; Figures 3, 4), similar results were observed. Controls achieved higher values than PwMS [F_(1,15)_ = 19.2, *µ*^2^ = 0.56, *p* < 0.001]. Controls achieved 83% normative values on the BWST, significantly higher than TBRS [62%; F _(1,15)_ = 13.9, *p* = 0.002, *µ*^2^ = 0.48]. PwMS performed similarly on the BWST (18%) vs. the TBRS (28%), both significantly below the BWST and TBRS achieved by Controls (Figure 3). When examining the MS disability subgroups, Control values were higher than both EDSS ≤2 (*p* = 0.001) and EDSS >2 groups (*p* < 0.001) on BWST but not using TBRS (Figure 4).

**Table 3:** Summary of metabolic parameters and criteria for achieving $\dot{V}O_{2\max}$ on TBRS and BWST.

Metabolic parameters and $\dot{V}O_{2\max}$ criteria	MS patients			Healthy controls
	All	EDSS >2	EDSS ≤2	
Metabolic parameters, TBRS				
$\dot{V}O_{2\max}$ (mL·min^−1^·kg^−1^)	26.53 ± 8.7	22.56 ± 10.1	30.50 ± 5.2	**34.32 ± 7.1** ^b^
RER (VCO_2_/VO_2_)	1.11 ± 0.1	1.06 ± 0.1	1.16 ± 0.1	1.12 ± 0.1
HR_max_ (bpm)	154 ± 30	140 ± 31	167 ± 25	170 ± 7.8
%$\dot{V}O_{2\max}$	**28 ± 23** ^c^	20 ± 24	36 ± 21	**62 ± 32** ^b^ ^,^ ^c^
%HR_max_	91.69 ± 17.8	88.95 ± 24.1	94.43 ± 10.8	98.66 ± 6.3
Metabolic parameters, BWST				
$\dot{V}O_{2\max}$ (mL·min^−1^·kg^−1^)	**24.24 ± 7.8** ^c^	**18.80 ± 5.4** ^d^	**29.67 ± 5.7** ^d^	**40.27 ± 7.6** ^b^ ^,^ ^c^ ^,^^d^
RER (VCO_2_/VO_2_)	1.04 ± 0.1	1.01 ± 0.0	1.07 ± 0.1	1.05 ± 0.1
HR_max_ (bpm)	152 ± 30	**132 ± 19** ^d^	172 ± 26	**177 ± 6.8** ^d^
%$\dot{V}O_{2\max}$	**18 ± 23** ^c^	**3 ± 4** ^d^	**32 ± 25** ^d^	**83 ± 20** ^b^ ^,^ ^c^ ^,^^d^
%HR_max_	90.38 ± 16.8	83.89 ± 20.1	96.88 ± 11.1	102.95 ± 5.5
Criterion for Achieving VO_2_^a^, TBRS				
RER (VCO_2_/VO_2_) ≥1.10	60	40	80	57
Within 10% of HR_max_ Predicted	50	40	60	86
$\dot{V}O_{2}$ plateau (150 mL·min^−1^)	40	80	0	14
Volitional Exhaustion	100	100	100	100
Achieved overall criteria for $\dot{V}O_{2\max}$ (≥2 of above criterion)	90	100	80	100
Criterion for achieving VO_2_^a^, BWST				
RER (VCO_2_/VO_2_) ≥1.10	20	0	40	43
Within 10% of HR_max_ predicted	50	20	80	100
$\dot{V}O_{2}$ Plateau (150 mL·min^−1^)	30	40	20	57
Volitional Exhaustion	50	40	60	100
Achieved overall criteria for $\dot{V}O_{2\max}$ (≥2 of above criterion)	60	40	80	100

Group Differences for MS Participants and Healthy Controls—EDSS, Expanded Disability Status Scale; MS, Multiple Sclerosis; RER, Respiratory Exchange Ratio; HR_max_, heart rate maximum(beats per minute, bpm); VCO_2_, volume of carbon dioxide; VO_2_, volume of oxygen; % $\dot{V}O_{2\max}$, % normative values for $\dot{V}O_{2\max}$; %HR_max_, % age-predicted heart rate maximum. Results are reported as mean ± standard deviation, unless otherwise indicated.

Bold values indicate statistically significant differences.

^a^ Criterion Results reported as percentage of participants who achieved the criterion.

^b^ Significant differences between TBRS and BWST (*p* < 0.05).

^c^ Significant differences between Controls and MS (*p* < 0.05).

^‡^Significant differences between the MS severity groups (EDSS <2, EDSS >2) and Control.

### HR_max_ and age-predicted HR_max_

In terms of HR_max_ values, there were no differences between MS and Controls [Group, F _(1,15)_ = 3.46, *p* = 0.08, µ^2^ = 0.18], between modalities [F _(1,15)_ = 0.57, *p* = 0.4, *µ*^2^ = 0.03], or interaction effect [F _(1,15)_ = 1.64, *p* = 0.2, *µ*^2^ = 0.09]. The mean values of HR_max_ were only different between Control and MS subgroup EDSS >2 (*p* = 0.002, 95% C.I. = 16.6, 73.6) on BWST but not on TBRS [F _(2,14)_ = 3.0, *p* = 0.08, *µ*^2^ = 0.3] (Table 2). In terms of age-predicted HR_max_, both PwMS and Controls reached 90% or greater (Table 3), {[F _(1, 15)_ = 2.23, *p* = 0.1, *µ*^2^ = 0.13]}, with no differences between the modalities [(TBRS vs., BWST), [F _(1, 15)_ = 0.49, *p* = 0.4, µ^2^ = 0.03]], or interaction effect {[F _(1, 15)_ = 1.75, *p* = 0.2, *µ*^2^ = 0.1]}. Also, there were no differences between the PwMS subgroups: EDSS ≤2, EDSS >2, and Control on either of BWST [F _(2, 14)_ = 3.26, *p* = 0.06, *µ*^2^ = 0.31], or TBRS [F _(2, 14)_ = 0.63, *p* = 0.5, *µ*^2^ = 0.08].

### Criteria for achieving a maximal cardiorespiratory fitness test

Overall, all Controls achieved the criteria for a $\dot{V}O_{2\max}$ test using both the TBRS and BWST (Table 4). Notably, all Control participants reached their predicted HR_max_ on the BWST, and 86% achieved this on the TBRS. All Controls reached volitional exhaustion using both modalities. Lastly, 57% reached a VO_2_ plateau using the BWST, whereas 14% reached a VO_2_ plateau using the TBRS. Five of the 10 PwMS were unable to continue on the BWST (due to leg weakness, pain or fatigue) which precluded the ability to determine whether VO_2_ plateau had been reached (Table 4). 90% of PwMS achieved the criteria for a $\dot{V}O_{2\max}$ test (i.e., two or more of the individual criterion) using the TBRS, whereas 60% achieved the criteria using the BWST. The RER criterion was reached by 60% of PwMS using the TBRS and 20% using the BWST. In the MS subgroup with greater disability (EDSS >2), more CPET criteria were reached using TBRS. These 5 participants reached 13/20 criteria on TBRS and only 5/20 on BWST.

**Table 4:** Criteria to satisfy the achievement of maximal aerobic capacity.

Group		RER (VCO_2_/VO_2_) ≥ 1.10		Within 10% of Age-Predicted HR_max_		VO_2_ Plateau (150mL*min^−1^)		RPE ≥7		Achieved criteria for $\dot{V}O_{2\max}$ [Yes or No (*n*) out of 4]	
	ID	TBRS	BWST	TBRS	BWST	TBRS	BWST	TBRS	BWST	TBRS	BWST
MS Patients (EDSS >2)	1	No	No	No	No	Yes	Undetermined^a^	Yes	No	Yes (2)	No (0)
	2	Yes	No	Yes	No	Yes	Undetermined^b^	Yes	No	Yes (4)	No (0)
	3	No	No	No	No	Yes	Undetermined^a^	Yes	No	Yes (2)	No (0)
	4	Yes	No	Yes	Yes	No	Yes	Yes	Yes	Yes (3)	Yes (3)
	5	No	No	No	No	Yes	Yes	Yes	Yes	Yes (2)	Yes (2)
MS Patients (EDSS ≤2)	6	Yes	No	No	Yes	No	Yes	Yes	Yes	Yes (2)	Yes (3)
	7	Yes	Yes	Yes	Yes	No	Undetermined^a^	Yes	No	Yes (3)	Yes (2)
	8	No	Yes	No	No	No	No	Yes	Yes	No (0)	Yes (2)
	9	Yes	No	Yes	Yes	No	Undetermined^a^	Yes	No	Yes (3)	No (1)
	10	Yes	No	Yes	Yes	No	No	Yes	Yes	Yes (3)	Yes (2)
Healthy controls	1	No	No	Yes	Yes	No	No	Yes	Yes	Yes (2)	Yes (2)
	2	No	No	Yes	Yes	Yes	Yes	Yes	Yes	Yes (3)	Yes (3)
	3	Yes	No	Yes	Yes	No	Yes	Yes	Yes	Yes (3)	Yes (3)
	4	Yes	Yes	No	Yes	No	Yes	Yes	Yes	Yes (2)	Yes (4)
	5	Yes	No	Yes	Yes	No	Yes	Yes	Yes	Yes (3)	Yes (3)
	6	Yes	Yes	Yes	Yes	No	No	Yes	Yes	Yes (3)	Yes (3)
	7	No	Yes	Yes	Yes	No	No	Yes	Yes	Yes (2)	Yes (3)

MS, multiple sclerosis; EDSS, expanded disability status scale; VCO_2_, volume of dioxide oxygen; VO_2_, volume of oxygen; RPE, rate of perceived exertion; Undetermined^a^, participant was unable to maintain the required speed (e.g., unable to walk faster). ID 1 could not continue due to fall. ID 3 had leg drags which made it too difficult to keep up with treadmill. ID 7 and 9 felt they needed to hold rails which became more difficult with increasing stages.

Undetermined^b^, participant stopped due to pain. ID 2 stopped due to cramping.

## Discussion

We undertook this study to optimize methods for achieving maximal CPET among PwMS with and without mobility disability, especially in longitudinal studies in which abilities, particularly walking ability, can change over time. We compared standard parameters between TBRS and BWST, including $\dot{V}O_{2\max}$, HR_max_, age- and sex-predicted % $\dot{V}O_{2\max}$, RER, RPE reached, age-predicted HR_max,_ and time to test completion. We also examined the criteria for reaching a maximal VO_2_ between modalities, including VO_2_ plateau (primary criterion), RER of at least 1.1, achieving at least 10% of age-predicted HR_max_, and a minimum of 7/10 RPE scale.

We identified three key findings. First, contrary to our hypotheses, Controls achieved higher $\dot{V}O_{2\max}$ and % $\dot{V}O_{2\max}$ on the BWST compared to the TBRS. We also expected that PwMS would achieve higher values on TBRS but in fact, PwMS performed similarly on both devices (Figures 1, 3). This likely contributed to significantly higher $\dot{V}O_{2\max}$ in Controls compared to PwMS only on BWST and not TBRS. Importantly, these modality-specific differences were not attributable to greater variability within the PwMS group, as variance in VO_2max_ was comparable between PwMS and Controls across both modalities. Secondly, while performing BWST, CPET was compromised among PwMS since half could not continue walking due to leg symptoms, which prevented the determination of VO_2_ plateau (Table 4). As for PwMS, 9/10 achieved the criteria for $\dot{V}O_{2\max}$ (i.e., two or more of the individual criterion) using the TBRS, whereas 6/10 achieved criteria using the BWST. All Controls achieved criteria for a maximum CPET on both devices (Table 4). Finally, a preliminary analysis of an MS subgroup having higher levels of mobility disability (*n* = 5) suggested that they achieved most of the criteria for a maximum CPET using TBRS [13/20 criteria achieved (five participants * four criteria) compared to CPET using BWST (5/20 criteria achieved; Table 4)]. The inability of participants with higher mobility disability to meet CPET criteria on the BWST likely contributed to significant differences between Controls and the PwMS with EDSS >2 (Figures 2, 4).

Consistent with prior work, Controls achieved lower VO_2max_ values and experienced greater difficulty meeting CPET criteria on the TBRS compared with the BWST (35). One potential explanation is that body weight support provided by the TBRS reduces whole-body weight-bearing demands, which may shift fatigue toward smaller, localized muscle groups in the upper or lower extremities (36, 37). According to the American College of Sports Medicine guidelines (22), such localized fatigue may limit peak oxygen uptake and contribute to the reported 5%–20% reduction in VO2_max_ values observed during supported or seated exercise modalities. This interpretation is further supported by studies reporting greater metabolic acidosis and higher lactate concentrations during exercise involving reduced active muscle mass, suggesting that local muscular fatigue may constrain aerobic power output (36, 37).

In PwMS, VO_2max_ values were similar between BWST and TBRS. This may reflect the interaction between modality-specific demands and mobility-related constraints. As suggested by Grover et al., in PwMS, BWST may induce greater impairment in the excitation-contraction coupling and higher muscle fatigue compared to TBRS (21). While BWST typically imposes higher metabolic demands, mobility-related limitations in PwMS, such as gait inefficiency, balance demands, fear of falling, and muscle fatigue, may limit treadmill VO_2max_, resulting in comparable VO_2max_ values across modalities.

Few studies disclose whether persons with neurological disability meet predefined criteria to satisfy reaching $\dot{V}O_{2\max}$ (38, 39). In one study, Mackay-Lyons and Makrides (20) reported CPET criteria performed 1-month post-stroke with 76% of patients achieving one or more of the $\dot{V}O_{2\max}$ criteria on BWST (20). Although 62% of the stroke patients achieved RER criteria on BWST (20), only 20% did in our patient sample. Similarly, there is a lack of information related to achieving CPET criteria in PwMS. Pilutti and group (19) compared TBRS and arm ergometry (EDSS=3.0), reporting that participants achieved similar $\dot{V}O_{2\max}$ on both devices (25.2 ± 6.8 vs. 22.5 ± 10.1 mL*min^−1^kg^−1^) (19), whether participants achieved the necessary criteria for $\dot{V}O_{2\max}$ was not reported. PwMS in the present study were more likely to reach criteria for a CPET on the TBRS which likely provided a more representative $\dot{V}O_{2\max}$ value. We propose that, although PwMS performed similarly on both devices (Figures 1, 3), the TBRS more reliably reflected supported assessment of CPET performance. Achieving criteria for testing PwMS should be noticed in future studies.

The plateau in VO_2_ stands out as a primary criterion in the measurement of aerobic capacity (40–42), and is the best evidence that a true $\dot{V}O_{2\max}$ is achieved (43). However, VO_2_ plateau (≤150 ml/min^−1^) has been criticized because of the lack of theoretical and statistical basis, as well as its insufficient specificity to the testing protocols (44). A significant variability in the percentage of subjects who showed a plateau in VO_2_ has also been reported (45). In this study, achieving VO_2_ plateau was the most challenging criterion to meet using BWST or TBRS for both PwMS and Controls. VO₂ plateau could not be determined in five PwMS during BWST testing due to the inability to maintain the required walking speed or test termination secondary to pain. Three participants (30% of our PwMS) achieved the VO_2_ plateau on BWST [more than the 17% reported previously in stroke (20) and similar to 34% of stroke patients tested on a semi-recumbent cycle ergometer (46)], while four participants (40% of our PwMS) achieved this criterion using TBRS. Some authors propose concerns that falling may prevent PwMS from pushing themselves hard enough on the BWST (16). Also with greater muscle fatigue reported on BWST (21), TBRS may be a better option for PwMS to achieve this criteria. It should be noted that a greater proportion of the Controls and PwMS with EDSS ≤2 achieved a VO_2_ plateau on BWST, whereas more PwMS with higher disability achieved a VO_2_ plateau on TBRS. This pattern may reflect that seated modalities may better expose VO_2_ plateaus in more disabled individuals by removing gait constraints, whereas ambulatory patients may more readily reach VO_2_ plateaus on treadmill protocols that impose a higher whole-body metabolic demand (47). These observations should be interpreted cautiously given the small sample size in the present work, and future studies should specifically compare testing modalities across MS severity levels.

Time to complete a CPET should be between 8 and 12 min (22). Most PwMS and all but one Control finished the test within 13 and 17 min, respectively, using the TBRS. The time ranged from 13 to 35 min using BWST across both groups, suggesting that TBRS was more efficient at obtaining $\dot{V}O_{2\max}$. Additionally, longer time on BWST may reflect lower-intensity, gait-limited pacing rather than greater aerobic workload. For participants with greater mobility impairment, BWST may constrain achievable intensity and compress VO_2max_ values, whereas TBRS probably enables self-paced, multi-limb effort that reveals the broader true range of cardiopulmonary capacity. Overall, older individuals or patients may require a longer time to achieve $\dot{V}O_{2\max}$ than healthy, trained, or active subjects (48). Grover et al. reported that PwMS had lower peak plantarflexor torque following 30 min BWST exercise compared to the same intensity and duration using TBRS (21). Participants in Grover's study also took a longer time to achieve their peak torques on BWST (21) which may relate to leg fatigue using BWST. Leg fatigue may limit the patient's perception of cardiopulmonary exertion on BWST (12, 16) lengthening the time taken to achieve $\dot{V}O_{2\max}$ on BWST compared to the recommended 8–12 min by Mezzani (49).

## Conclusion

PwMS achieved similar $\dot{V}O_{2\max}$ and % $\dot{V}O_{2\max}$ values on the BWST and TBRS, whereas healthy Controls achieved higher values using BWST. A greater proportion of PwMS met predefined criteria for achieving a maximal CPET using the TBRS compared with the BWST. Participants with higher disability (EDSS >2) more consistently achieved CPET criteria on the TBRS, while controls achieved criteria on both modalities. These findings suggest that TBRS may be a feasible option for mobility-adapted CPET in PwMS. Selection of CPET modality should consider disability level and task demands when assessing aerobic capacity in clinical and research settings.

## Limitations

This study has several limitations. The sample size was small and derived from a previously conducted studies, which limits statistical power and generalizability. Therefore, caution should be taken in extrapolating the results to all PwMS. Participants were recruited from a single referral center, and controls were recruited by convenience sampling, which may reduce representativeness. In addition, some participants with MS were unable to complete BWST testing, limiting the determination of VO₂ plateau and constraining modality comparisons. Although the within-subject crossover design strengthens modality comparisons, findings should be interpreted as exploratory. Although disability stratification was performed using an EDSS cut-off, the inclusion of participants across a broad disability range remains a limitation and constrains mechanistic interpretation. Future studies with larger, prospectively recruited cohorts are needed to confirm these results across the MS disability spectrum.

## Data Availability

The raw data supporting the conclusions of this article will be made available by the authors, without undue reservation.

## Supplementary material

The Supplementary Materialfor this article can be found online at: https://www.frontiersin.org/articles/10.3389/fresc.2026.1731215/full#supplementary-material
