# Supplementary material for: Body-weight supported treadmill or total body recumbent stepper for mobility-adapted cardiopulmonary exercise testing in multiple sclerosis patients with varying disability
Source: Front Rehabil Sci. 2026 Mar 5;7:1731215. doi: 10.3389/fresc.2026.1731215 (PMC12999428; doi:10.3389/fresc.2026.1731215)
Supplement: Supplementary file 1 [file Table1.docx]

# **Supplement A.**

The STROBE Reporting Checklist

|  | Item Description | Location (or reason for not reporting) |
| --- | --- | --- |
| Title and abstract |  |  |
| 1a. Indicate the study’s design | Indicate the study’s design with a commonly used term in the title or the abstract. | Abstract, Line three |
| 1b. Abstract | Provide in the abstract an informative and balanced summary of what was done and what was found. | Abstract (Intervention- Main outcome and Results) |
| Introduction |  |  |
| 2. Background / rationale | Explain the scientific background and rationale for the investigation being reported. | Introduction- paragraphs one and two |
| 3. Objectives | State specific objectives, including any prespecified hypotheses. | Introduction- Last paragraph |
| Methods |  |  |
| 4. Study design | Present key elements of study design early in the paper. | Methods-second paragraph |
| 5. Setting | Describe the setting, locations, and relevant dates, including periods of recruitment, exposure, follow-up, and data collection. | Methods-second paragraph |
| 6a. Eligibility criteria | Cohort study: Give the eligibility criteria, and the sources and methods of selection of participants. Describe methods of follow-up. Case-control study: Give the eligibility criteria, and the sources and methods of case ascertainment and control selection. Give the rationale for the choice of cases and controls. Cross-sectional study: Give the eligibility criteria, and the sources and methods of selection of participants. | Methods- First paragraph |
| 6b. Matching criteria | Cohort study: For matched studies, give matching criteria and number of exposed and unexposed. Case-control study: For matched studies, give matching criteria and the number of controls per case. | Methods- First paragraph |
| 7. Variables | Clearly define all outcomes, exposures, predictors, potential confounders, and effect modifiers. Give diagnostic criteria, if applicable. | (Methods, par.6)-The primary outcome variables were VO₂max, %VO₂max, maximum heart rate (HRmax), age-predicted HRmax, Respiratory Exchange Ratio (RER), and achievement of maximal CPET according to established criteria (RER ≥ 1.1, attainment of age-predicted HRmax, and/or VO₂ plateau). The main exposure variable was exercise test modality (BWST vs TBRS), with participant group (PwMS vs controls) as a secondary variable. Potential confounders were reduced by randomized order of test administration and control matching. |
| 8. Data sources / measurement | For each variable of interest give sources of data and details of methods of assessment (measurement). Describe comparability of assessment methods if there is more than one group. | (Methods, par.6)- All data were obtained through direct cardiopulmonary exercise testing (CPET) performed by trained staff using standardized protocols. (Methods, Experimental Design)- Participants completed CPET on two devices (BWST and TBRS) in randomized order.  (Methods, par.5)-VO₂max, %VO₂max, HRmax, age-predicted HRmax, and RER were measured using an indirect calibrated calorimetry. (Methods, par.6)-Maximal CPET achievement was determined by accepted physiological criteria (RER ≥ 1.1, attainment of age-predicted HRmax, and/or VO₂ plateau). (Methods, par1)-Demographic data (age, sex) were self-reported and verified at enrollment.  (Methods, par1)- Clinical status of PwMS (disease stability, MoCA score, ambulatory ability) was assessed by the study neurologist. (Methods, par 3&4)-Measurement procedures were identical for PwMS and controls, and across both test modalities. |
| 9. Bias | Describe any efforts to address potential sources of bias. | (Methods, par.1)- To reduce selection bias, PwMS were recruited from a clinical referral pathway with predefined inclusion/exclusion criteria, and controls were age- and sex-matched.  (Methods, par 3&4)-To limit measurement bias, the same calibrated metabolic cart and standardized CPET protocol were used across both test modalities and participant groups. (Methods, par.2)- The order of device testing (BWST vs TBRS) was randomized for each participant to minimize order and learning effects. (Methods, par.6)-Assessors were trained in CPET procedures and adhered to consistent testing protocols.  (Methods, par.1)-Potential confounding from sex and age was reduced through control matching. |
| 10. Study size | Explain how the study size was arrived at. | Methods, par.1 |
| 11. Quantitative variables | Explain how quantitative variables were handled in the analyses. If applicable, describe which groupings were chosen, and why. | Methods, statistical analyses: line 4 |
| 12a. Statistical methods | Describe all statistical methods, including those used to control for confounding. | Methods, Statistical Analyses |
| 12b. Statistical methods – subgroups and interactions | Describe any methods used to examine subgroups and interactions. | Methods, Statistical Analyses |
| 12c. Statistical methods – missing data | Explain how missing data were addressed. | No missing data |
| 12di. Statistical methods – loss to follow-up | Cohort study: If applicable, describe how loss to follow-up was addressed. | N/A |
| 12dii. Statistical methods – matching cases and controls | Case-control study: If applicable, explain how matching of cases and controls was addressed. | N/A |
| 12diii. Statistical methods – sampling strategy | Cross-sectional study: If applicable, describe analytical methods taking account of sampling strategy. | N/A |
| 12e. Statistical methods – sensitivity analyses | Describe any sensitivity analyses. | No sensitivity analyses were performed. |
| Results |  |  |
| 13a. Participant numbers | Report the numbers of individuals at each stage of the study—e.g., numbers potentially eligible, examined for eligibility, confirmed eligible, included in the study, completing follow-up, and analyzed; Consider use of a flow diagram. | N/A |
| 13b. Participants – non-participation | Give reasons for non-participation at each stage. | N/A |
| 13c. Participants – flow diagram | Consider use of a flow diagram. | N/A |
| 14a. Descriptive data – participant characteristics | Give characteristics of study participants (e.g., demographic, clinical, social) and information on exposures and potential confounders. Present the information in a table. | Table 1 |
| 14b. Descriptive data – missing data | Indicate the number of participants with missing data for each variable of interest. | N/A |
| 14c. Descriptive data – follow-up time | Cohort study: Summarize follow-up time—e.g., average and total amount. | N/A |
| 15. Outcome data | Cohort study: Report numbers of outcome events or summary measures over time. Case-control study: Report numbers in each exposure category, or summary measures of exposure. Cross-sectional study: Report numbers of outcome events or summary measures. | Table 2 |
| 16a. Main results | Give unadjusted estimates and, if applicable, confounder-adjusted estimates and their precision (e.g., 95% confidence intervals). Make clear which confounders were adjusted for and why they were included. | Table 2 |
| 16b. Main results – category boundaries | Report category boundaries when continuous variables were categorised. | Table 4 |
| 16c. Main results – risk | If relevant, consider translating estimates of relative risk into absolute risk for a meaningful time period. | N/A |
| 17. Other analyses | Report other analyses done—e.g., analyses of subgroups and interactions, and sensitivity analyses. | Table 3 |
| Discussion |  |  |
| 18. Key results | Summarise key results with reference to study objectives. | Discussion, par.2 |
| 19. Limitations | Discuss limitations of the study, taking into account sources of potential bias or imprecision. Discuss both direction and magnitude of any potential bias. | Discussion, par.7: Study Limitations |
| 20. Interpretation | Give a cautious overall interpretation considering objectives, limitations, multiplicity of analyses, results from similar studies, and other relevant evidence. | Discussion, par.8: Conclutions |
| 21. Generalisability | Discuss the generalisability (external validity) of the study results. | Discussion, par.8: Conclusions |
| Other information |  |  |
| 22. Funding | Give the source of funding and the role of the funders for the present study and, if applicable, for the original study on which the present article is based. | Funding |
